# Evolving insights in blood-based liquid biopsies for prostate cancer interrogation

**DOI:** 10.18632/oncoscience.592

**Published:** 2023-11-30

**Authors:** R. Daniel Bonfil, Ghaith Al-Eyd

**Affiliations:** ^1^Department of Medical Education, Dr. Kiran C. Patel College of Allopathic Medicine, Nova Southeastern University, Fort Lauderdale, FL 33328, USA

**Keywords:** prostate cancer, liquid biopsy, circulating tumor cells (CTC), circulating tumor DNA (ctDNA), exosomes

## Abstract

During the last decade, blood sampling of cancer patients aimed at analyzing the presence of cells, membrane-bound vesicles, or molecules released by primary tumors or metastatic growths emerged as an alternative to traditional tissue biopsies. The advent of this minimally invasive approach, known as blood-based liquid biopsy, began to play a pivotal role in the management of diverse cancers, establishing itself as a vital component of precision medicine. Here, we discuss three blood-based liquid biopsies, namely circulating tumor cells (CTCs), circulating tumor DNA (ctDNA) and tumor-derived exosomes, as they relate to prostate cancer (PCa) management. The advances achieved in the molecular characterization of these types of liquid biopsies and their potential to predict recurrence, improve responses to certain treatments, and evaluate prognosis, in PCa patients, are highlighted herein. While there is currently full clinical validation for only one CTC-based and one ctDNA-based liquid biopsy for patients with metastatic castration-resistant PCa, the adoption of additional methods is anticipated as they undergo standardization and achieve analytical and clinical validation. Advantages and disadvantages of different blood-based liquid biopsy approaches in the context of PCa are outlined herein, while also considering potential synergies through combinatory strategies.

## INTRODUCTION

Worldwide, prostate cancer (PCa) ranks as one of the most frequently diagnosed cancers among males, contributing significantly to cancer-related mortality [1]. In the United Sates, 288,300 new cases of PCa are estimated for 2023, resulting in approximately 34,700 deaths [2]. Currently, the 5-year relative survival rate for patients with localized and regional PCa is nearly 100%. However, despite significant therapeutic advances in recent years, once PCa metastasizes to distant organs, it ultimately relapses due to the development of treatment resistance, particularly castration-resistance. Consequently, there is a substantial decline in the 5-year relative survival rate, reducing it to around 30% and leading to an invariably fatal condition [3]. To improve the clinical outcome for these patients, it is imperative to advance the understanding of the molecular and cellular mechanisms that PCa uses to evade therapy. Once these mechanisms are adequately explained, new tools can be identified and validated to provide information about the likelihood of disease progression and response to a particular therapy, thus enhancing personalized treatment decisions.

Although prostate biopsy stands as the singular gold standard procedure for a definitive diagnosis of PCa, it provides a snapshot of limited locations within the primary tumor at a specific time, rather than capturing the heterogeneity present in different areas of the neoplasm [4]. Moreover, the incorporation of metastatic site biopsies aimed at depicting a more comprehensive landscape of advanced PCa, may be challenging due to limitations associated with identifying and accessing widespread lesions [5].

Over the last decade, the analysis of various patient-derived biological fluids, commonly referred to as *liquid biopsies*, has emerged as an alternative for cancer researchers and oncologists to explore non-invasive techniques that may overcome some of the limitations associated with tissue biopsies. Since the growth and spread of solid tumors depend on an adequate blood supply, there has been a growing focus on blood-based liquid biopsies, as they allow repeated sampling from patients and facilitate the monitoring of cancer progression and the assessment of response to treatment in real time [6]. Furthermore, since the primary tumor and metastatic lesions continuously shed cells and molecular components into the bloodstream [5, 7], blood-based liquid biopsies provide a valuable approach for analyzing different biomarkers that might carry genomic, epigenomic, transcriptomic, proteomic, or metabolomic tumor information.

In this research perspective, we present a comprehensive overview of the recent advances related to the clinical significance of blood-based liquid biopsies in PCa, with a primary emphasis placed on key biomarkers such as circulating tumor cells (CTCs), circulating tumor DNA (ctDNA), and exosomes.

### Circulating tumor cells

Cells sloughed off from invasive primary tumors that enter the venous circulation are referred to as CTCs and represent a transitional phase in the progression towards metastasis, as only a few of those cells survive and are able to extravasate in distant organs [8]. CTCs circulate in peripheral blood as individual cells or cell clusters, and it is likely that the latter exhibit higher metastatic potential and correlate with a shorter overall survival (OS) time for patients [9]. CTCs – which usually survive for a few hours – sometimes are found in the bloodstream of patients who had their primary tumors resected a significant time earlier, strongly suggesting that they can also be shed from micrometastases [10].

The isolation of CTCs poses technical challenges due to their extremely low concentration in circulation, with an estimated ratio of approximately one CTC per billion normal blood cells in patients with advanced cancer [11]. More or less sophisticated methodologies have been developed during the last few decades to isolate and identify CTCs on the basis of their physical properties (cell size, density, deformability, dielectric properties), cell surface markers (antigens expressed by tumor cells but not by blood cells), or through the straightforward process of lysing red blood cells (reviewed in [8, 12–14]).

**Figure 1:**
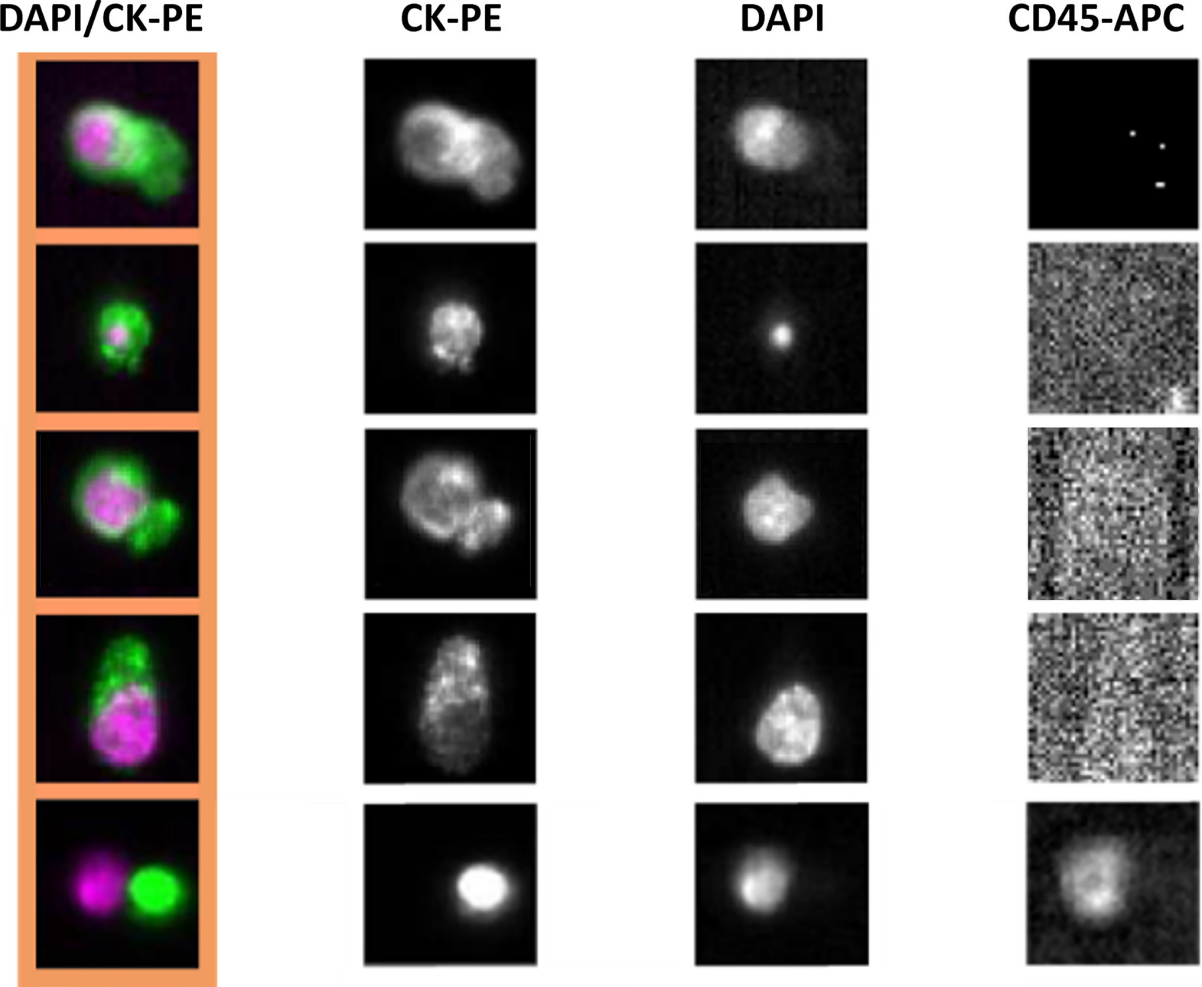
CTCs captured and identified using the CellSearch^®^ system. Image gallery obtained after processing 7.5 mL of whole blood obtained from a mCRPC patient with bone metastasis. Four CTCs were enumerated based on the presence of cells with a diameter >4 μm with DAPI-intact nuclei; expression of cytokeratins (CKs) 8, 18, and 19 (CK-PE, anti-CK-Phycoerythrin) - specific for epithelial cells, and lack of expression of CD45 (CD45-APC, anti-CD45-Allophycocyanin) - specific for leukocytes. Combined fluorescence channels (composite view in left column), and images for each of the separate fluorescence channels are shown.

To date, the CellSearch^®^ system (Menarini Silicon Biosystems Inc., Bologna, Italy) stands as the sole technology cleared by the U.S. Food and Drug Administration (FDA) for the isolation, identification, and enumeration of CTCs from patients with PCa [15]. This automated platform employs a ferrofluid-coupled antibody to magnetically capture CTCs derived from carcinomas through targeting the epithelial cell adhesion molecule (EpCAM) which is not expressed by blood cells. Following the enrichment step, fluorescence-labeled antibodies against cytokeratins (CKs) 8, 18, and 19 (epithelial antigens), CD45 (a leukocyte marker), and DAPI (a nuclear stain) are employed to facilitate the visualization and enumeration of CTCs present in 7.5 mL of whole blood (Figure 1), as described elsewhere [16]. CTC counts obtained using the CellSearch^®^ system emerged as an independent and superior predictor of OS in metastatic castration-resistant prostate cancer (mCRPC) patients compared to prostate serum antigen (PSA) levels, with a cut-off level of 5 or more CTCs per 7.5 mL of blood consistently associated with reduced OS [15, 17]. Additional studies have confirmed the clinical utility of CTC enumeration using the CellSearch^®^ system as a biomarker of disease progression in mCRPC patients [18–20].

During the malignant progression of carcinomas, including PCa, neoplastic epithelial cells can undergo an epithelial-to-mesenchymal transition (EMT), which has been proposed to contribute to invasion, resistance to apoptosis, and metastatic dissemination [21, 22]. Moreover, EMT entails the downregulation of epithelial markers, including E-cadherin, cytokeratin, and EpCAM, while simultaneously exhibiting an upregulation of mesenchymal markers, such as vimentin and N-cadherin, which reflect the transition from an epithelial state to a mesenchymal state [23]. Therefore, although some CTCs that co-express both epithelial and mesenchymal markers (partial EMT) can still be isolated using enrichment systems that rely on expression of EpCAM or other epithelial markers [24, 25], others that are more mesenchymal-like may be missed. In that sense, the use of technologies that do not rely on the detection of antigens expressed by CTCs would be advantageous. One such system is Parsortix^®^ PC1 (ANGLE North America, Inc., King of Prussia, PA), a microfluidic platform that enables the capture and retrieval of intact single CTCs and CTC clusters, based on the less deformable nature and larger size of CTCs compared to other blood cells, regardless of their expression of epithelial, mesenchymal, or other cell surface markers [26]. Last year, Parsortix^®^ was FDA-cleared for use in the isolation of circulating metastatic breast cancer cells for liquid biopsy testing [27]. This system has also been assayed with blood obtained from CRPC patients, demonstrating its ability to accurately quantify CTCs exhibiting both epithelial and mesenchymal immunophenotypes and its potential for a range of molecular and functional studies [28, 29]. Studies are currently underway to assess the clinical significance of this platform in large cohorts of PCa patients and its potential for FDA evaluation.

While the enumeration of CTCs can shed light on PCa progression and patient outcomes, it is expected that the molecular characterization of these cells may uncover crucial information for a more comprehensive assessment of the tumor’s behavior and potential response to specific therapies. For instance, expression of the androgen receptor (AR) splice variant 7 (AR-V7) has been associated with reduced response to AR signaling inhibitors (ARSI) enzalutamide and abiraterone, as well as with shorter progression-free survival (PFS) and OS in patients with mCRPC [30, 31]. Another noteworthy aspect about gene expression analysis in CTCs is its potential as surrogate samples for metastatic PCa lesions, which are infrequently biopsied or challenging to access. Studies conducted by both our team and others on small cohorts of PCa patients with metastatic bone disease have shown some level of concordance between gene expression profiles in CTCs and matching skeletal metastases [24, 32]. Moreover, another group performed a mutational characterization of 11 mCRPC patients using whole-genome amplification (WGA) analysis at the single cell level using three different strategies to capture CTCs with epithelial, mesenchymal, or hybrid phenotypes. The study revealed some recurrent somatic mutations shared among CTCs, mainly with epithelial phenotype, that in many cases correlated with matched metastatic lesions of the patients [33]. Recently, the feasibility of using the label-free CTC enrichment method Parsortix^®^ for transcriptome profiling of both single and aggregate forms of epithelial and mesenchymal CTCs was confirmed in a cohort of mCRPC patients, suggesting that a carefully chosen gene panel with high expression in PCa cells and low expression in leukocytes could serve as a predictive tool for therapy response [34]. While these results are promising, further studies involving larger patient populations and diverse CTC enrichment platforms are required to better define the genomic and transcriptomic landscapes of metastatic PCa using CTCs and evaluate their clinical utility. Regarding proteomic analysis of CTCs, researchers are currently investigating cutting-edge technologies like imaging mass cytometry (IMC) that, when combined with high-definition single-cell analysis (HD-SCA), has demonstrated the capability to detect up to 40 proteins in CTCs obtained from a patient with mCRPC [35]. Despite this finding, further in-depth studies are required to determine whether this multiplex protein detection technique can provide valuable clinical information for identifying predictive and/or prognostic biomarkers from CTCs isolated from PCa patients.

**Figure 2:**
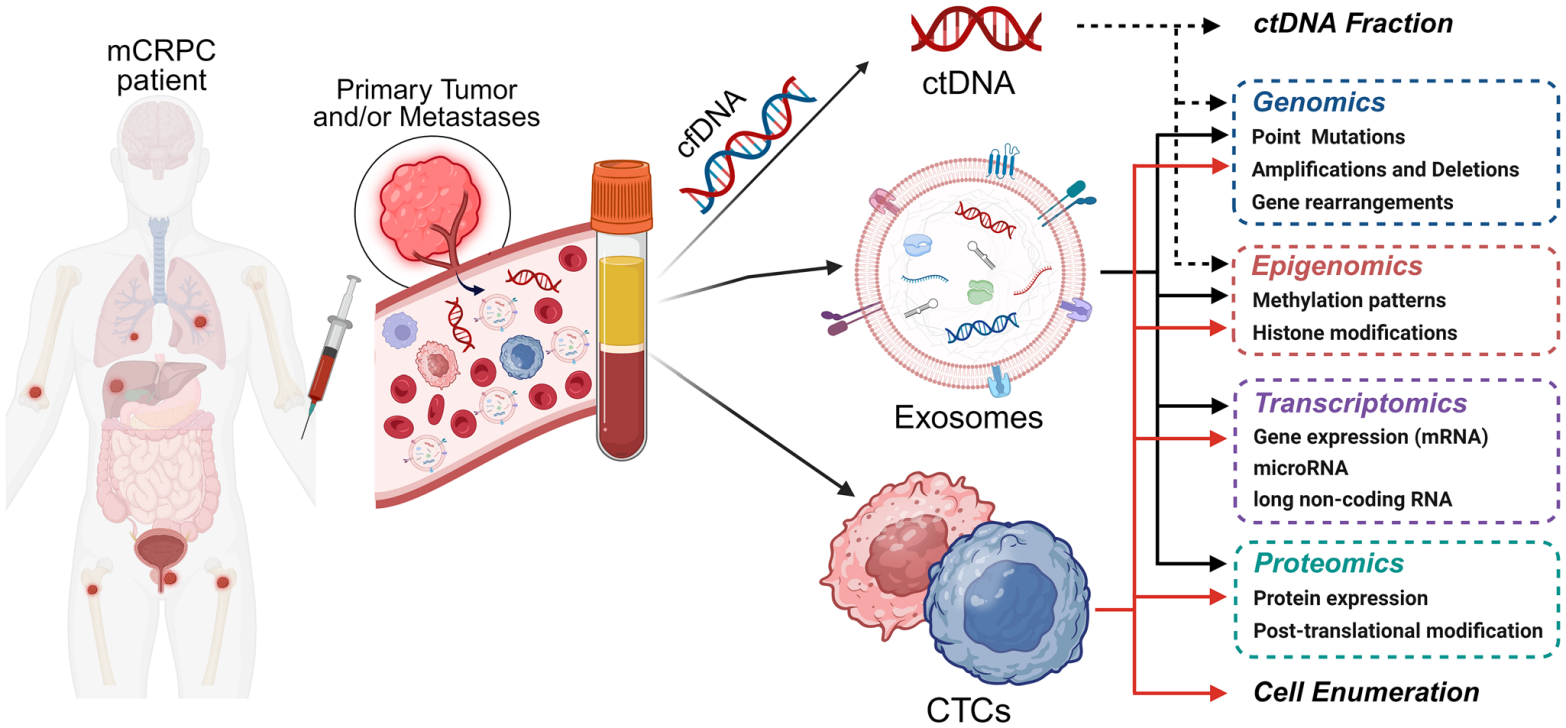
Analysis of blood-based liquid biopsies in prostate cancer. Examples of molecular analyses enabled by the isolation of circulating tumor cells (CTCs), circulating tumor DNA (ctDNA), and tumor-derived exosomes, are illustrated. Abbreviations: mCRPC: metastatic castration-resistant prostate cancer; cfDNA: cell-free circulating DNA.

In spite of some limitations, CTCs hold clinical significance at multiple levels in PCa management, not only offering prognostic and predictive insights but also crucial DNA, RNA, and protein data that can inform targeted approaches to address metastatic disease (Figure 2).

### Circulating tumor DNA

The existence of cell-free circulating DNA (cfDNA), believed to be released into the bloodstream through apoptosis or necrosis, was first reported by Mandel and Metais in 1948 [36]. Decades later, it was discovered that patients with various cancers displayed an abundance of cfDNAs as compared to healthy individuals [37, 38]. Compared to cfDNA from non-neoplastic cells, which usually ranges from 160 to 180 base pairs (bp), ctDNA has been reported to be shorter (132–145 bp) [39] and to contain specific genomic alterations, such as point mutations, aberrant copy numbers, and methylation patterns, commonly present in genes involved in oncogenesis [40]. Furthermore, levels of ctDNA have been found to correlate with tumor burden and to change in response to treatment (reviewed in [41]).

Patients with solid cancers often present a very low fraction of ctDNA within the cfDNA (less than 0.1%), which poses significant technical challenges for its detection and analysis [42]. Several targeted techniques, such as droplet digital polymerase chain reaction (ddPCR) and beads, emulsion, amplification, and magnetics (BEAMing), are used to detect somatic point mutations in ctDNA with very high sensitivity [43, 44]. While valuable, these techniques may overlook the tumor heterogeneity represented in ctDNA due to their restricted ability to analyze a limited number of mutations at a time. As a result, untargeted approaches like whole-genome sequencing (WGS) and whole-exome sequencing (WES), which consist of panels of 500 to 600 genes, can be used for identifying point mutations or somatic copy number alterations (SCNAs). However, these untargeted methods require higher ctDNA concentrations due to their lower sensitivity [43, 44].

As of now, several ctDNA-based assays have obtained FDA approval as companion diagnostic (CDx) assays for screening specific gene alterations to guide the use of targeted therapies, mainly in certain patients with advanced non-small cell lung cancer and breast cancers [45, 46]. As for PCa, Tukachinsly et al. conducted a comprehensive study using the FoundationOne Liquid^™^ assay, which assesses 70 genes, to test the plasma from over 3,000 patients with mCRPC [47]. They were able not only to find ctDNA in 94% of the patients studied, but also a high level of concordance between alterations in various genes, such as *AR*, *TP53*, and *BRCA 1/2*, identified using both ctDNA and tissue-based comprehensive genomic profiling (CGP) [47]. The FDA has indeed approved FoundationOne Liquid^™^ as a CDx for identifying patients with mCRPC who have germline or somatic mutations in *BRCA1* and/or *BRCA2* genes who could benefit from for treatment with the Poly (ADP-ribose) polymerase (PARP) inhibitors olaparib and rucaparib [48].

Several studies have shown an association between elevated baseline cfDNA concentrations and ctDNA% values (the proportion of ctDNA in total cfDNA) and reduced PFS and OS in mCRPC patients treated with ARSI (e.g., enzalutamide, abiraterone) and chemotherapy (e.g., docetaxel, cabazitaxel) [49–51]. Furthermore, it has been reported that patients with mCRPC patients who exhibit persistent ctDNA levels after four weeks of initial treatment with ARSI are at a higher risk of developing acquired resistance within six months of commencing treatment, resulting in shorter PFS and OS outcomes [51]. Nevertheless, even though these findings are noteworthy, their true predictive potential is still under evaluation, as there are currently no clinically relevant thresholds established for cfDNA concentration and ctDNA% in this context [52].

In advanced stage PCa, ctDNA presents a promising alternative to traditional tissue biomarker analysis. This is supported by studies of ctDNA and matched metastatic tissue biopsies that revealed a strong concordance in several gene alterations, including *AR* gene amplification and inactivating mutations in tumor suppressor genes (e.g., *TP53*, *PTEN*, *RB1*, *BRCA2*) [53, 54], suggesting that ctDNA analyses may be useful for molecular stratification of PCa patients with metastatic disease for more precise prognostic and predictive purposes. Moreover, genomic alterations in *AR* that can drive resistance to ARSI and are commonly found in mCRPC, such as copy number amplification, have also been identified in ctDNA and are associated with worse clinical outcomes [55].

Analysis of ctDNA has also shown promise as a non-invasive approach for detecting neuroendocrine prostate cancer (NEPC), an aggressive PCa that can emerge from prostate adenocarcinoma as a mechanism of treatment resistance or may arise *de novo* [56, 57]. Clinical features, such as rapid disease progression, elevated PSA levels, and widespread metastases, might raise suspicion for NEPC. However, for an accurate diagnosis of NEPC, the presence of neuroendocrine markers (chromogranin and synaptophysin by immunohistochemistry) needs to be confirmed in metastatic tumor biopsies [58], which can be difficult to obtain. As an alternative to this invasive approach, Beltran et al. have studied WES and whole-genome bisulfite sequencing of ctDNA and matching metastatic tumor biopsies, which showed a high concordance in genomic alterations and methylation profiles [59]. Overall, specific genomic alterations (*TP53*, *RB1*, *CYLD*, and *AR* loss) and epigenomic changes (e.g., *ASXL3* and *SPDEF* hypermethylation, and *INSM1* and *CDH2* hypomethylation) reflective of metastatic lesions were identified in ctDNA from patients with NEPC [59]. These results support the potential use of ctDNA genomic and epigenomic patterns to recognize transformation to NEPC using a noninvasive approach and to allow earlier initiation of chemotherapeutic treatment in these patients.

Although, unlike other blood-based liquid biopsies, ctDNA does not provide transcriptomic or proteomic information (Figure 2), the study of its concentration values and genomic and epigenomic profiles has shown potential clinical utility. These parameters can be used to assess progression of PCa with a prognostic value, to monitor response to treatment in mCRPC patients and risk of developing acquired resistance (predictive value), and to effectively diagnose aggressive variants of PCa such as NEPC with high accuracy.

The use of ctDNA-based assays for identifying specific actionable genomic alterations in mCRPC has been approved by the FDA as a CDx that guides treatment decisions, enabling a more personalized and targeted approach to therapy.

### Exosomes

Both normal and cancer cells can release extracellular vesicles (EVs) that are surrounded by a lipid bilayer membrane and carry a diverse cargo of DNA, RNA, lipids, or proteins [60]. EV biogenesis occurs through invagination of the plasma membrane and formation of intracytoplasmic multivesicular bodies that then merge with the cell membrane to release EVs into the extracellular space and disperse them throughout body fluids [61]. Exosomes are a subset of EVs with diameters typically ranging from 40 to 160 nm that have been reported to play an important role as intercellular messengers carrying different molecules to nearby or distant cells affecting physiological as well as many pathological processes, such as tumor progression and metastasis [60, 61]. For example, tumor-derived exosomes can disrupt the extracellular matrix by affecting the adhesion of cells through integrins and facilitating the degradation of collagen and fibronectin, thereby promoting invasion and metastasis. Different studies have also shown that tumor-derived exosomes can facilitate angiogenesis through the transfer of different cargo molecules, such as miRNAs, that modulate the expression of vascular endothelial growth factor (VEGF) or Hypoxia-inducible Factor 1α (HIF-1α), which is an important regulator of VEGF [62, 63]. Furthermore, exosomes present within the tumor microenvironment (TME) can contribute to the emergence of resistance to cancer therapy by orchestrating a wide range of signaling interactions among diverse cell types within the TME that enable cancer cells to better adapt and evade treatment (reviewed in [64]). In addition, the presence of programmed death-ligand 1 (PD-L1), an inhibitory checkpoint molecule, on the surface of tumor-derived exosomes has been reported to promote immune evasion of cancer cells by suppressing the function of cytotoxic T cells [65].

Exosomes have emerged as a compelling avenue for liquid biopsies due to a range of advantages: (a) their abundance in body fluids (~10^9^ particles/mL of plasma), which simplifies isolation, (b) they arise from viable cells, encompassing substantial representative tissue information, and (c) their robust lipid bilayer structure that makes them stable carriers of DNA, RNA, proteins, and lipids from the cell of origin, enabling extended specimen storage and yielding enhanced testing opportunities [61].

While a consistent technique for isolating a pure population of exosomes from fluids is still lacking, some current strategies being used include ultracentrifugation, density-gradient separation, ultrafiltration, and affinity-based capture techniques that employ antibodies or specific ligands targeting exosome surface markers [66]. Following isolation, diverse analysis methods are employed to profile the cargo of exosomes. These include immunoblotting and mass spectrometry for proteomics, RNA sequencing to characterize microRNAs (miRNAs), messenger RNAs (mRNAs), and long non-coding RNAs (lncRNAs), as well as DNA sequencing [64]. However, it is crucial to be cautious when evaluating the content profile of exosomes, as research has shown that the composition analysis of exosomes from the same cell type can vary depending on the isolation method used [67].

In a comparative study involving patients with advanced cancers, blood-derived exosomal DNA was found to exhibit greater efficacy as a cancer biomarker than ctDNA [68]. However, genomic studies on plasma-derived exosomes specifically obtained from PCa patients have not been documented thus far, possibly because of their significantly smaller amounts of DNA compared to larger extracellular vesicles originating from the same patients [69]. In contrast, various studies have been conducted to investigate the involvement of exosomal RNAs in the development and progression of PCa. For instance, research has shown that *AR-V7* mRNA can be sensitively detected through ddPCR in plasma-derived exosomes [70], and that its detection has proven to hold predictive value for patients with metastatic PCa who are experiencing progression on androgen deprivation therapy [70, 71]. Furthermore, the expression of exosomal *TUBB3* mRNA – which encodes the Class III β-tubulin protein – was found to correlate with unfavorable PFS in mCRPC patients who underwent first-line abiraterone treatment, suggesting that it could potentially serve as an indicator to identify individuals less likely to respond positively to this treatment [72]. Nevertheless, miRNAs have received greater attention due to their exceptionally stable form that remains safeguarded against endogenous RNase activity, and because they are the most abundant RNA species found within plasma-derived exosomes [73]. Using quantitative reverse transcription polymerase chain reaction (qRT-PCR), Bryant et al. discovered that among 742 miRNAs studied in plasma samples taken from 78 PCa patients and 28 healthy control individuals, the levels of 11 miRNAs were significantly higher in the former group. Moreover, in a subsequent analysis performed in two independent cohorts of patients, the authors found that miR-141 and miR-375 were significantly increased in serum-derived exosomes from patients with metastatic PCa compared to those with non-recurrent PCa [74]. In another study using data for 375 known miRNAs, Huang et al. conducted RNA sequencing on a screening cohort of 23 CRPC patients, aiming to identify plasma-derived exosomal miRNAs associated with OS. Candidate miRNAs were then assessed in a follow-up cohort of 100 CRPC patients using qPCR to evaluate prognosis. The study found that patients with high levels of miR-375 and miR-1290 were significantly associated with poor OS [73], highlighting their prognostic potential. Additional studies conducted on smaller patient cohorts have corroborated the elevated expression of the previously mentioned blood-derived exosomal miRNAs, as well as others, and their association with progression, staging, and outcomes of patients with PCa (reviewed in [75]). Although promising, the validation of some of these findings in larger independent cohorts is necessary to establish the clinical applicability of blood-derived exosomal miRNAs in PCa patients.

While proteomic analyses appear to offer more advantages than transcriptomic studies, as they directly identify the presence of functional proteins rather than miRNA or upstream mRNA precursors, up to date only a few of those analyses have been performed in plasma or serum of PCa patients. For instance, in a study involving exosomes obtained from the sera of 36 PCa patients with bone metastases – comprising eight untreated individuals, eight under primary androgen deprivation therapy (ADT), and 20 with CRPC – a mass spectrometry analysis identified a total of 787 unique proteins. Among these, the expression levels of six proteins were found to be increased in CRPC patients compared to those undergoing ADT. Notably, only one protein, namely actinin-4 (ACTN4), showed significantly higher expression in CRPC than in ADT patients [76]. This discovery is of particular interest due to ACTN4’s known association with the core actin structures of invadopodia in carcinoma cells [77], and the finding that *ACTN4* gene knockdown in DU 145 (a well-know PCa cell line) leads to significant decrease of *in vitro* invasiveness [76].

In another recently published study, an untargeted proteomics analysis was conducted from a total of 938 proteins identified on plasma exosomal proteins collected from tumor-free individuals and PCa and CRPC patients. The expression levels of specific exosomal proteins were subsequently confirmed within the three groups. This validation revealed that leucine-rich α-2 glycoprotein 1 (LRG1) and inter-α-trypsin inhibitor heavy chain H3 (ITIH3) exhibited significantly higher levels in the CRPC group compared to the PCa group [78]. Previous studies have described the role of LRG1 in angiogenesis [79]. Furthermore, through mass spectrometry analysis of sera with reduced levels of abundant proteins, it was shown that heightened LRG1 levels are detrimental for patients newly diagnosed with high-risk or metastatic PCa [80], thereby corroborating the aforementioned findings. Regarding ITIH3, it is important to approach the drawn conclusions with caution, considering that a prior study across various human solid tumors, including PCa, has reported downregulation of *ITIH3* expression in cancer profiling arrays containing spotted tumor cDNAs when compared with matching normal tissue [81]. Taken together, these findings lend support to the hypothesis that the expression of certain serum-derived exosomal proteins could function as both prognostic and predictive markers, as well as potential therapeutic targets for PCa. However, further validation in larger longitudinal cohort studies is required to confirm these observations.

Besides blood samples, exosome isolation from urine can be employed to stratify patients with high-grade PCa (Grade Group 2) who are aged ≥50 years and have PSA levels between 2 and 10 ng/mL, which is considered an intermediate range that is not definitively indicative of a specific condition affecting the prostate. Indeed, the ExoDx^™^Prostate IntelloScore (EPI) test (Exosome Diagnostics, Inc., Waltham, MA, USA) has been certified by the FDA to discriminate indolent from clinically significant PCa, based on the measurement in urine of three PCa-specific exosome-derived RNA biomarkers (*ERG*, *PCA3*, and *SPDEF*) [82]. Nevertheless, the analysis of urine-derived exosomes might hold less significance for PCa patients with disseminated disease, in whom exosomes sourced from plasma could offer valuable prognostic and predictive insights. This is particularly pertinent given the distant occurrence of metastases and the likelihood that some patients within this group have undergone radical prostatectomy.

Therefore, exosomes present a promising array of clinical applications in PCa patient management due to their inherent stability, abundant presence, and capacity to carry representative native tissue information. Hence, genomic, epigenomic, transcriptomic, and proteomic analyses of exosomes (Figure 2) may soon become feasible diagnostic, prognostic and/or predictive tools for patients with PCa.

## CONCLUSION

The advent of liquid biopsies is causing a paradigm shift in the approach to diagnosing, monitoring, and treating various cancers, including PCa. This less conventional type of biopsies not only is minimally invasive as compared with traditional tissue biopsies, but also can be repeated over multiple points in time allowing for real-time monitoring of cancer progression and treatment response. This, in turn, enables us to stratify PCa patients more effectively and tailor personalized therapeutic strategies.

Until now, the CellSearch^®^ system (for enumeration of EpCAM-positive CTCs in CRPC) and the FoundationOne Liquid^™^ CDx (for profiling *BRCA1* and/or *BRCA2* mutations in ctDNA in mCRPC patients) stand as the sole FDA-approved or cleared blood-based liquid biopsy tests for PCa. This can be partly attributed to the stringent standards set for test approval to guarantee accuracy and reliability, and the extensive trials required for clinical validation. An illustration of the latter is the Parsortix^®^ system, which has already been approved for use in patients with metastatic breast cancer but is still undergoing evaluation in extensive cohorts of PCa patients to ascertain its validity for clinical use in the context of this disease.

The aim of this Research Perspective is to provide current and pertinent information on three blood-based liquid biopsies in PCa, namely CTCs, ctDNA and exosomes. Each of them presents strengths and limitations that we summarized in Table 1. Although, unlike CTCs and ctDNAs, no exosome test has been validated to date, advancements in this type of liquid biopsy hold promise considering the high concentrations of PCa-derived exosomes found in blood and their comprehensive molecular profiles amenable for analysis.

**Table 1:** Summary of potential applications of blood-based liquid biopsies in the management of prostate cancer

Type of liquid biopsy	Advantages	Limitations
**CTCs**	Cell enumeration has prognostic value in mCRPC patients Potential as predictive biomarkers for patient stratification based on molecular characterization Potential use for establishment of cell lines and functional testing One FDA-approved platform is available	Found in low concentrations Isolation technical challenges Detection of some CTC subpopulations is dependent on the enrichment strategy used Maintenance of viability is challenging (optimization of CTC culture conditions is needed)
**ctDNA**	More stable than RNA or cells Levels correlate with tumor burden and response to treatment Genomic profiles show high concordance with those from solid tumor biopsies Potential for tracking emergence of aggressive PCa subtypes, such as NEPC One FDA-approved companion diagnostic assay	Low concentration hinders detection and analysis Potential background noise from non-tumor cfDNA Derived from apoptotic or dead cells No transcriptomic or proteomic information
**Exosomes**	High abundance in blood Derived from living cells Cargo molecules remain stable, protected from degradation due to their lipid bilayer structure Potential use as prognostic and predictive markers based on molecular characterization	Consistent high efficiency and purity isolation methods still lacking Molecular profiling is dependent on the isolation method used Scarcity of clinical data No FDA-approved system is currently available

Abbreviations: cfDNA: cell-free circulating DNA; CTCs: circulating tumor cells; ctDNA: circulating tumor DNA; FDA: Food and Drug Administration; mCRPC: metastatic castration-resistant prostate cancer; NEPC: neuroendocrine prostate cancer; PCa: prostate cancer.

Additional investigation is essential to determine the clinical validity and utility of diverse blood-derived liquid biopsies in PCa patients. Securing clinical qualification for both existing and novel tests demands not only prior confirmation of their sensitivity and specificity but also necessitates their standardization for widespread implementation and the establishment of reference values. Moreover, exploring multiparametric analysis by combining strategies for liquid biopsy from a single blood sample could be undertaken to determine if resolution can be enhanced, thereby expanding the range of clinical utility in PCa patients. We anticipate that, with the assistance of artificial intelligence based on pre-established parameters, the utilization of blood-based liquid biopsies will soon enhance the stratification of PCa patients and facilitate timely therapeutic decision-making.
